# Bibliography

**Published:** 1904-04

**Authors:** 


### Bibliography.

A Compend of Dental Pathology and Dental Medicine. Containing the most noteworthy points upon the subjects of interest to dental students. By Geo. W. Warren, A.M., D.D.S., Professor of Principles and Practice of Operative Dentistry, Pennsylvania College of Dental Surgery, Philadelphia. Fourth edition. Illustrated. P. Blakiston's Son & Co., Philadelphia.

When a compend has reached a fourth edition, it demonstrates conclusively that it has met a need existing in undergraduate study.

There have been many improvements in this small volume since it was reviewed in an earlier edition in this journal, and, for brief statements, the chapter on Development of the Teeth, and the following sub-chapters, on the Structure and Anatomy of the Teeth, are well condensed, principally from Broomell and Peirce.

It seems to the writer that it is impossible to consider, in the space usually given to subjects in compends, the important topics connected with dental pathology and therapeutics, hence criticism must be left for more elaborate productions. This, however, must be said of it, that the author has been measurably successful in managing his pathology so far as he has gone. In his efforts to condense he has omitted much that should have been noticed, and has not detailed treatment sufficiently to make it valuable to the average student. Forty-five small pages is the limit of Dental Pathology. This is followed by "Dental Medicine," to which seventy-three pages are devoted. An Appendix on "Emergencies" close the book. For a pocket reference the book has a special value to students, but if it is desired to know the subjects thoroughly, more extensive works must be consulted.

Organic and Physiologic Chemistry. A Manual for Students and Practitioners. By Alexius McGlannan, M.D., Associate Professor of Physiologic Chemistry, Instructor in Clinical Laboratory, College of Physicians and Surgeons, Baltimore, Md. Series edited by V. C. Pedersen, A.M., M.D. Illustrated. Lea Brothers & Co., Philadelphia and New York.

The author says of his work in this manual, that "The purpose . . . is to select from the immense mass of knowledge accumulated in its department such facts as are of essential importance to medical students and practitioners, and to present them in a style facilitating comprehension and recollection."

To give a general idea of this manual and its comprehensive treatment of subjects, the contents, under general heads, in Part I., Organic Chemistry, is given. Chapter 1. General Principles. 2. The Constitution, Decomposition, and Classification of Organic Compounds. 3. The Hydrocarbons. 4. The Alcohols. 5. The Aldehydes. 6. The Organic Acids. 7. The Polybasic and Hydroxy Acids. 8. The Ethers, Esters, Fats, Oils. 9. Nitrogen in Organic Compounds. 10. The Carbohydrates. 11. The Benzene or Aromatic Series. 12. Benzene Derivatives containing Nitrogen. 13. The Proteins. 14. The Methods for Quantitative Analysis.

This is followed by Physiologic Chemistry in seven chapters, followed by an Appendix on Physical and Chemical data, Weights and Measures, Metric and Apothecaries' System Equivalents, etc. Similar to the others of the Pedersen Series, this manual is quite superior to books that ordinarily are published under that name, and are proportionably valuable to the student as a pocket reference.

